# Stakeholders’ perception on the organization of chronic care: a SWOT analysis to draft avenues for health care reforms

**DOI:** 10.1186/1472-6963-14-179

**Published:** 2014-04-18

**Authors:** Thérèse Van Durme, Jean Macq, Sibyl Anthierens, Linda Symons, Olivier Schmitz, Dominique Paulus, Koen Van den Heede, Roy Remmen

**Affiliations:** 1 Institute of Health and Society, Université catholique de Louvain, Clos Chapelle-aux-Champs, Brussels, Belgium; 2 Centre for General Practice, Universiteit Antwerpen, Campus Drie Eiken, Wilrijk, Belgium; 3 Belgian Health Care Knowledge Centre, Kruidtuinlaan, Brussels, Belgium

**Keywords:** Health care reform, Long-term care, Health services, Primary care

## Abstract

**Background:**

Adequate care for individuals living with chronic illnesses calls for a healthcare system redesign, moving from acute, disease-centered to patient-centered models. The aim of this study was to identify Belgian stakeholders’ perceptions on the strengths, weaknesses, opportunities and threats of the healthcare system for people with chronic diseases in Belgium.

**Methods:**

Four focus groups were held with stakeholders from the micro and meso level, in addition to two interviews with stakeholders who could not attend the focus group sessions. Data collection and the discussion were based on the Chronic Care model. Thematic analysis of the transcripts allowed for the identification of the strengths, weaknesses, opportunities and threats of the current health care system with focus on chronic care.

**Results:**

Informants stressed the overall good quality of the acute health care system and the level of reimbursement of care as an important strength of the current system. In contrast, the lack of integration of care was identified as one of the biggest weaknesses of today’s health care system, along with the unclear definitions of the roles and functions of health professionals involved in care processes. Patient education to support self-management exists for patients with diabetes and/or terminal kidney failure but not for those living with other or multiple chronic conditions. The current overall fee-for-service system is a barrier to integrated care, as are the lack of incentives for integrated care. Attending multidisciplinary meetings, for example, is underfinanced to date. Finally, clinical information systems lack interoperability, which further impedes the information flow across settings and disciplines.

**Conclusion:**

Our study’s methods allowed for the identification of problematic domains in the health system for people living with chronic conditions. These findings provided useful insights surrounding perceived priorities. This methodology may inspire other countries faced with the challenge of drafting reforms to tackle the issue of chronic care.

## Background

The World Health Organization (WHO) defines a chronic disease as requiring, “…ongoing management over a period of years or decades” [1]. The term traditionally refers to diabetes, cardiovascular and renal diseases, mental disorders, cancer, COPD (chronic obstructive pulmonary disease) and diseases like HIV/AIDS. Chronic diseases are responsible for over three quarters of the global burden of disease in industrialized countries [1,2]. Higher life expectancy, changing lifestyles, and improved medical technology are all factors that increase their prevalence [2]. The provision of high quality and accessible care is a challenge for the health care system and the society as a whole [3].

Recent publications from the European Commission and the European Union Policy Forum summarize the burden of chronic diseases [4,5]: 40% of the population in Europe above the age of 15 is reported to have a chronic disease; such diseases are responsible for 86% of deaths. An additional problem is that chronic diseases rarely come alone [6]. Chronically ill persons often suffer from several problems: fifty to seventy percent of individuals over the age of 70 have at least two disorders [7]. The current health systems of many OECD countries tend to focus on acute conditions [8]. However, people with chronic diseases have broader needs like more intensive medical, psychological, psychosocial, social and spiritual support [9].

Some national health systems, such as the Netherlands or Denmark, have developed national plans to face the challenge of chronic care [10,11]. Common elements of these plans include the drafting of a national framework for the provision of chronic care, the promotion of integrated care, the implementation of a disease management processes and the set-up of personal health care plans [12]. In line with these international developments, the Belgian Minister of Health & Social Affairs asked for the development of a national position paper on the future of chronic care in Belgium [13,14]. This position paper is based on various data sources: (1) publications from international organizations; (2) national plans of four countries; (3) a review of the literature on patient empowerment and on new functions for healthcare professionals; (4) a description of Belgian initiatives, and (5) an extensive consultation process of active stakeholders in the Belgian health care system.

The current article describes a qualitative study on stakeholders’ perceptions, carried out within the context of the development of the national position paper mentioned before. The aim of this study was to describe how stakeholders perceive the strengths, weaknesses, opportunities and threats (SWOT) of the Belgian health care system in relation to the care of chronically ill people and to identify new avenues for a national healthcare reform.

Belgium is a small country in Europe. The part of the GDP spent on health care is within the European average. Belgian health care is characterized by free entrance to primary, secondary and tertiary care facilities. General practitioners (GPs) do not play the role of gatekeeper, and referrals are not required. Physicians are most often paid on a ‘fee-for-service’ basis and are self-employed. Patients have an obligatory medical insurance by which some medical services are reimbursed. Out of pocket payment accounts for approximately 25% of health expenses. In Belgium, almost 99% of the population is covered by a compulsory health insurance. In primary care, nursing care is provided by large scale organisations (salaried nurses) or small scale independent providers (self-employed). The Federal Government has provided incentives to lower competition between providers, targeting more integration by implementing regional multidisciplinary organisations. In recent years the number of hospital beds has been steadily declining [8].

## Methods

A SWOT analysis was performed among key informants in spring 2012. This method was previously used in policy research to systematically analyze organizations’ environments [15]. If used correctly, SWOT-analyses can offer policy makers a sound basis for strategy development and formulation.

### Sample recruitment and selection

A purposive sampling technique targeted key informants involved in chronic care delivery at the micro and meso level in the French and Dutch speaking communities of Belgium. Micro and meso levels of health care are based on the WHO-definition [16].The meso level comprises the health system at both the local and the organizational level, such as hospitals. The main roles include the provision of health services, as well as the coordination, supervision and training of service providers. The micro level includes the citizens, local providers and services, local authorities and the interactions between them. Inclusion of both groups was considered crucial to obtain individuals’ perceptions, and information on interactions and on organizational levels of primary care practices.

Stakeholders were identified through formal and informal networking, reflecting both the views of patients as well as the disciplines/functions involved in the care for people with chronic conditions. Selection aimed to have a maximum of 12 people per group so as to represent the different professional profiles (i.e. physicians, social workers, nurses, pharmacists and representatives of patients and informal caregivers) and the various domains of activity (i.e. direct care, management, coordination). If invited people were unavailable or refused, people with a similar profile were invited in a second wave of invitations.

### Data collection and analysis

Stakeholders from the micro and meso levels were interviewed to provide a SWOT analysis of the Belgian health care system. They were invited to four separate sessions, two per language group (French and Dutch). The rationale behind the different language groups was to (1) enhance fluent communication within groups sharing the same language and (2) have a coherent discussion within groups as the regulations regarding accreditation of health care institutions are different in the French and Dutch speaking parts of Belgium. French- and Dutch-speaking stakeholders of the Brussels’ and German-speaking Region were also included.

The interview guide was structured following the domains of the adjusted Chronic Care Model, a framework for the effective organization of chronic care [13,17]. This step was completed with two additional semi-structured individual interviews with informants who were not able to attend the focus groups.

Data were collected primarily by the means of focus groups. Focus groups are particularly well-suited for our purposes as they have multivalent functions: (1) a *pedagogical* function, involving collective engagement to promote dialogue and to achieve higher levels of understanding, (2) a *political* function, to transform the conditions of existence for particular stakeholders and (3) a *research* function, which can certainly be considered a main element here, since it allows for *“…the generation of rich, complex, nuanced and even contradictory accounts of how people ascribe meaning to and interpret their experience with an eye toward how these accounts might be used to affect social policy and social change.”* (p.546) [18]. Focus groups and interviews were audio-taped after written informed consent was obtained. For focus groups, audio-tapes were supplemented by field notes of two observers. Researchers coded the data from the two focus groups independently, conducting a thematic content analysis [18] and a meeting was held to discuss the final themes with the whole research team. Based on these emerging themes, a SWOT analysis was performed and organized according to the domains of the Chronic Care model. Data from the interviews were used to validate the findings of the focus groups, as no additional themes emerged from the interviews.

### Ethical approval

The Ethics Committee of the University of Antwerp approved this study (B300201214276).

## Results

In April and May 2012, four focus groups, two per language group, were organized with stakeholders from the micro and meso levels of chronic care. The 33 participants are presented in Table 1. Each focus group lasted two hours.

**Table 1:** Focus Group Participants and Interviewees (i.e. professionals who could not attend the focus groups)

**Representatives of …**	**Focus group members (n)**	**Inter-viewed (n)**	**Region**
			**D = Dutch-speaking Region**
			**F = French-speaking Region**
			**B = Brussels’ Region**
			**Ger = German-speaking Region**
Patient organizations	3		D, F, Ger
Informal caregiver organisations	2		D, F
Pharmacist organisations	2	1	D, F
GP organisations	6		D, F, B
Nursing home managers	3		D, B
Specialists	2	1	D
Primary care nurses	7		D, F, B
Regional care organisations	5		D, F, B, Ger
Advanced nurse practitioner (hospital-based)	3		D, F, B

Key results of the SWOT analysis of all focus groups were grouped into six themes listed below.

### Theme 1: continuum of care within and between lines: a call for coordination

Participants highlighted the lack of integration of care as one of the largest weaknesses of today’s health care system. Patients with chronic conditions often ‘navigate’ between various health and social care providers. This situation calls for better coordination in primary care and for the organization of seamless care among secondary care providers.

(Nurse, 2nd focus group) “*There is a tension when we work together with different care providers around a single patient: you need a team attitude. You have to know the boundaries of your work. With how many people can you work without losing control?”*

Multidisciplinary coordination of care was seen as crucial, especially for complex situations. Formal coordination of care, probably by the means of a case manager, could help to identify resources for patients living with complex conditions, particularly for problems that extend beyond their medical conditions.

#### Strengths

The informants mentioned a number of initiatives which are currently promoting seamless care, e.g. discharge policies from hospitals for older patients (external liaison) and federally/regionally funded coordination centers that not only coordinate care at the primary care level but also between hospitals and primary care.

#### Weaknesses

Coordination initiatives are fragmented and overlap for many reasons: (1) coordination initiatives are either *organization-centered* (e.g. home care organization offering a care package including nursing care, family and household support, meals on wheels, etc.), or *single disease-centered* (e.g. care pathways for diabetes or chronic kidney failure). (2) GPs’ integration in these structures is almost seen as incidental. Even if everyone agrees that the GPs’ role in primary care is crucial, there are many barriers preventing him/her from playing this role. For instance, GPs are expected to makes certain decisions, e.g. about the right moment for hospital discharge, but when it comes to assess the preparedness of a home situation, he/she does not necessarily have the required information to make a decision:

(Nurse, 2nd focus group) *“We see that GPs are overburdened, they become less accessible, but they are still given the central role, even though they are not in the middle of information when there is a complex situation. Decisions are not always made by persons who are best informed.”*

(3) Care and help organizations are structured in many ‘silos’, such as health care versus support care, private organizations versus public-funded organizations, primary care versus institutional care, silos per discipline, etc. and each of these entities does not know about the functioning of the other entity (4). In many situations, no one is formally designated as responsible for care coordination, so designating a dedicated person for this function, such as a case manager, seems the most promising avenue.

Opportunities were not mentioned in this domain.

#### Threats

(1) Patients’ freedom of choice of care provider, a major concept in the Belgian system, was seen as a threat to care coordination, as these care providers can be hired by different organizations. These care providers come from different structures or are independent and therefore do not share the same values, visions, clinical information tools, etc. (2) Informants expressed a form of hierarchy between physicians; hospital specialist did not consider primary care GPs’ opinion as valuable, nor did they ask for previous treatments:

(GP, 2nd focus group): *“They (i.e. teaching hospitals) think that the world stops at the limits of the hospital and they do not even consider the time between the discharge of the patient one year before, for vascular surgery, and his admission one year later. The specialist did not even contact me to know about the current treatment.”*

### Theme 2: new definition of the roles of care professionals and their training

The second important theme was related to the clear definition of the roles and functions of health professionals. This topic is related to the previous theme of coordinated care.

#### Strengths

In hospitals, disease-specific teams prepare the patient for discharge, e.g. for chronic heart failure or transplantation. In these teams, roles and functions are clearly defined; professionals are highly specialized and skilled within their (sub)discipline.

#### Weaknesses

These roles are sometimes well-defined within single-disease pathways in hospitals but this is not the case at home, especially for patients with multimorbidity. There is therefore a need for generalist care providers, as opposed to specialist care providers, who are trained to take up the role of coordination of care, such as case managers. Training for this function does not exist yet in Belgium. Moreover, most of the GPs, seen as crucial to the coordination of care, are working in solo practices and their training focuses on acute and specialized care:

(GP, 3rd focus group) “*There is a problem with the training of GPs, which focuses on acute and specialized care. I think that GP care should be more present in medical faculties (…). Only in general practice is one aware of the fact that there is more to it than disease: the home and the family must be accounted for, but this is not part of our training.”*

Finally, the current legislation makes task delegation difficult because there are no financial incentives in a fee-for-service system.

No opportunities and threats were mentioned for this domain.

### Theme 3: patient empowerment and support for informal caregivers

Two key issues emerged in the focus groups: (a) patient education to support self-management and (b) support activities for informal caregivers in order to enable them to keep on caregiving in adequate conditions.

(Patients and informal caregivers’ association, 2nd focus group) “*If we hope that a patient decides for his/herself, we have to be sure that he/she understands the options. We should enable him/her to meet his/her peers, and we should provide information about support groups. This is not systematically offered by professionals.”*

#### Strengths

The necessity to empower patients is clear to care providers. Health and patient education are being professionalized through better training of care providers; patient empowerment is central to patient education. This means that patients are better able to express their priorities and preferences, which may not be the same as the ones of the care providers:

(Network coordinator, 2nd focus group) *“We often see that medical doctors’ priorities are different from patient priorities. The thing that interested the patient in the first place was his housing and food problems, because the only food he had in his fridge was a soda can, to treat hypoglycemia”.*

The education role by nurses is enhanced by the work of patient associations, who bring an added value to education by the means of peer education. Secondly, some organizations provide telemonitoring and this is also seen as a support for care management.

Two strengths were highlighted in relation to informal caregivers as (1) there is a law project under way which will establish an official status for informal caregivers, so that the time spent in informal care is taken into consideration for the calculation of their pension, or social security,. (2) A minimum wage is supplied for informal home caregivers in Flanders.

#### Weaknesses

Informants stressed the fact that patient education is mostly (single)-disease centered. Patients lack accessibility to relevant information because (1) the legal framework organizing reimbursement systems and care delivery is too complex; (2) the role of patient organizations is too weak; and (3) information is not well-organized and is obsolete. As a result, patients (a) miss information and therefore lack access to tools to make informed choices and (b) are rarely included in the quality evaluation of the services provided, which may, in turn, have an impact on the service.

Concerning the informal caregiver, support and respite structures are insufficient, as are incentives to suspend or reduce their professional activities. Overall, informal caregivers are not considered partners in care.

(Patient association, 3rd focus group) *“It often happens that, in cancer patients, communication with health care providers is difficult. Informal caregivers do not find their place, and are caught between the beneficiary and the health care providers. They are afraid to express themselves, afraid to be ill-perceived and cumbersome.”*

No opportunities were mentioned for this domain.

#### Threats

Participants stressed that the coordination role is often taken on by a professional despite the willingness of the patient or his/her informal caregiver to coordinate his/her own care.

(Patient association, 1st focus group) *“The informal caregiver is sometimes willing to coordinate. However, professionals take on this [coordinating] role, which seems logical, because it’s part of their training. But this means that it is such a knot to undo, that it discourages families to ask for help from the coordination services.”*

### Theme 4: the payment system: an obstacle to integrated care

The Belgian health care system mainly relies on fee-for-service in ambulatory care but other payment mechanisms coexist (capitation, lump sums for specific services). The fee-for-service system is perceived as a barrier for task delegation and for the provision of integrated care by a multidisciplinary team.

#### Strengths

Some stakeholders suggested that a capitation system would offer an added value for the provision of care to patients with complex chronic conditions.

(GP, 4th focus group) “*Palliative care is not financed by a fee-for-service system. This is a dream for a GP who works with these palliative teams because they have time. Working with nurses in a fee-for-service system is unbearable because they are always under stress.”*

Capitation is already the case in some medical centers; *nursing care facilities for dependent patients use* payment per episode through care pathways and lump sum reimbursement systems.

#### Weaknesses

The coexistence of different payment systems is also a weakness, because (1) in a given area, similar providers use different types of payment systems and also (2) because the system is hard to understand for care beneficiaries and even for care providers. Moreover, the predominant system is fee-for-service, which may lead to (a) a push for quantity and professional stress and (b) difficult task delegation, because the professional who is delegating is not paid if another care provider delivers the health care. Thirdly, some care provision is not reimbursed; e.g. monitoring of vital parameters is not properly financed, the social worker is not reimbursed, etc. Finally, when capitation systems are used, difficulties arise because (1) most payment systems are disease-oriented (e.g.: diabetes); (2) reimbursement criteria are ill-adapted (e.g. patient dialysis is reimbursed if the patient lives at home, but not if he/she undergoes auto-dialysis in a nursing home). (3) GPs’ “Global Medical File”, an incentive for comprehensive care by GPs, is underused.

No opportunities and threats were mentioned for this domain.

### Theme 5: clinical information systems

Important changes in the clinical information systems are expected to have an impact on the organization of care for chronic patients.

#### Strengths

The progressive generalization of electronic patient records within settings and the ICT federal platform of social security (eHealth) have allowed for shared information. Input of electronic data should help obtain quality (self-) assessment of care provision. Moreover, linking clinical files with built-in algorithms should support clinical decision-making.

#### Weaknesses

First of all, in many cases, communication around a single patient living at home occurs by the means of paper documents, which are considered suboptimal. Secondly, indices show a lack of an overall, coherent vision of the health care system. This is evident when looking at the myriad of pilot experiments, each using its own tools, leading, for instance, to incompatibility between software. Thirdly, the lack of uniform care language hinders information sharing across settings. As a consequence, there is currently a lack of aggregated data for quality management purposes. Informants also stressed the lack of built-in algorithms for assessing the incompatibility between drug prescriptions. Fourth, information about these tools and systems is not available. For instance, care providers in the study had heard about the ICT federal platform for the first time during our focus groups.

#### Opportunities

(Social worker, 1st focus group) *“It should be made possible for the patient to access his data, in order to claim the care or service provision to which he is entitled: preferential reimbursement rates, etc.”*

#### Threats

Professionals and patients expressed fear regarding the security and privacy of sensitive data, leading to reluctance to use shared electronic data files. Professionals fear (external) quality control, leading to intrusion in clinical in clinical decision-making from the authorities.

(GP, 3rd focus group, about electronic patient records) “*This is very positive, but raises some ethical questions, because the patient might be unwilling to share his/her data. Important safety procedures should be set up.”*

### Theme 6: accessible care

Accessible care is a key issue for people with chronic care needs (i.e. timely care that is provided by the right professional, in the right setting and at an affordable price).

(Director of a nursing home, 4th focus group): “*There is a shortage of accommodation for chronic care patients. There are too few housing facilities; we are regularly confronted with demands from people we are unable to even enroll on a waiting list.”*

#### Strengths

Participants stated that overall, care is timely as well as financially and geographically accessible. Because emergency rooms are open 24/7, where payment is delayed, in most cases, a hospitalization can be an easy solution for a crisis situation at home (e.g. acute overburdening of the informal caregiver). In addition, emergency telephone lines for off-hour services within the primary care practice appear to be effective. Aside from this overarching network of reimbursed, accessible healthcare, some local initiatives provide support for patients with chronic conditions, who are at risk of being rejected from insurance companies.

Weaknesses of the system include long delays (1) for specialist consultations, especially in remote areas (e.g. ophthalmologists) and (2) nursing homes, who work with long waiting lists. At the same time, financial accessibility is hindered for middle class chronic patients, who do not have access to non-health care or family aids: they are both too wealthy to benefit from social security funds and too poor to pay for it. Finally, the payment system does not account for the real care needs and provisions (see above). For instance, at-home care services are not affordable for the majority of chronic patients.

No opportunities and threats were mentioned for this domain.

## Discussion

This study illustrates the importance to involve stakeholders in future reforms in a health care system. A robust qualitative methodology allowed for the collection of important elements for future reforms. Stakeholders’ perspectives highlighted major issues in relation to the organization of the Belgian health care system and its capacity to answer to the needs of the patient with chronic disease. In particular, the stakeholders stressed the importance of the four domains for improvements listed below. These domains should be addressed simultaneously, in order to achieve better chronic care.

Firstly, priority should be given to the organization of care at the patient level [19]. Proposals for improvement in this domain included the intervention of a case manager, as this was seen as the most obvious solution to address the issue of fragmented care. Indeed, amongst other roles, one of the main roles of the case manager is to coordinate the care adequately around a single patient. For many chronic patients, comprehensive care is becoming too complex for a GP to handle during routine care [20]. Indeed, biomedical needs are often linked to psychological and social needs [21]. The role and function of a case manager should therefore encompass the latter and should be well-defined [22]. This position requires specific training [23], legal and cultural changes [24], and adequate financing [25]. In Belgium, these conditions are not yet adequately addressed and urgent attention of all players in the field is needed. In addition, the role of the case manager is rather undefined at present. Should they have medical background (i.e. nurses) and what should be their optimal level of expertise and education? Stakeholders agreed that the case manager should have sufficient expertise himself, or within a team of case managers, to be able to perform a comprehensive assessment of a whole situation and be able to monitor all health-related determinants to achieve meaningful goals for the patient and his informal caregiver. The best profile for this, in the Belgian situation, was a nurse case manager, with a specific training for chronic care management at the Master’s level. Furthermore, one must decide at what level in the health system case managers should work. In some countries, these case managers work at the level of primary care practices [24]. However, Belgium currently lacks these practices to a large extent; many of them are independently working general practitioners [8].

Secondly, shared electronic files should allow adequate data transmission between professionals and multiple disciplines, and also between primary and secondary care [26]. Legislation issues (i.e. privacy) need to be tackled [26]. Moreover, information systems in this country are highly diverse with poor interoperability [27], which may lead to communication gaps between providers and subsequently to fragmented care [26]. In addition, evidence demonstrates the importance of built-in guidelines for supporting clinical evidence-based decision-making [26]. Policy makers in Belgium seem to be willing to support the integration of several preexisting tools that were developed for specific users. This is particularly challenging in Belgium as ICT networks are often different for French and Dutch communities and also between all disciplines of primary and secondary care. It was acknowledged that there is an urgent need to share platforms (like eHealth), compatible with comprehensive geriatric assessments (BelRAI, a Belgian version of the InterRAI) [29], with links to validated guidelines for Belgium (EBMPracticeNet) [28], and with high level security access and the necessary legal privacy clearances. However, many barriers exist, one of them being the low ICT literacy of (elderly) health care providers and the inability of the programs to properly communicate with each other.

Thirdly, empowering patients should be addressed by the means of participation and self-determination in health decision making [30]. However, results show that this informed decision making is hindered by the difficult access to relevant information about the available financial and material resources, as the Belgian system is very complex both to beneficiaries and even to care providers. Alas, this complexity is even expected to increase as the Belgian health care system will undergo an important reform, by the means of transfer of skills from the Federal to the Regional level in the forthcoming months.

Fourthly, adequate payment systems are needed to support comprehensive care. The prominent payment model in this country is pay for service [31]. This model does not foster the implementation of task sharing and delegation [32]. Capitation systems in this country can be adopted in primary care practices but this is, to date, not very popular as only minorities of less than 5 percent of GPs adopt this schema. It has been suggested that capitation based payment may result in lower than average care [31]. However, previous research has shown that primary care practices that adopted the capitation schema showed better adherence to guidelines and provided better preventive medicine [33,34].

### Limitations of the analysis

Stakeholders’ consultation can help identify strengths and weaknesses for health care reforms in a specific context. We tried to ensure that all important groups be consulted e.g. patients, informal caregivers and professionals working on the field (at the micro and meso level).

A number of steps in this study aimed at decreasing as much as possible the subjectivity that might have influenced the results and interpretations:

– *Multidisciplinary research team.* The researchers had diverse clinical and cultural backgrounds, from the French-speaking parts and from the Dutch speaking parts of the country. Their skill-mix allowed for adequate reflection on the views expressed during the interviews.

– *Informants with diverse experiences.* Informants were selected from diverse domains, functions, professions, in order to reflect the views and concerns of a variety of disciplines and interest groups. Of special importance was to include informants of different Regions, as the Regions differ in many aspects, possibly impacting chronic care. For instance, the German-speaking is now moving into a higher level of integrated care and the Brussels’ Region because their population is more multi-ethnic and shows higher levels of poverty.

– *Information gathering process.* A key issue was the choice of challenging propositions whilst minimizing the possible subjectivity during this phase. Still the approach allowed us to build statements on the situation as experienced by the stakeholders on the field.

Few opportunities and threats were identified by the stakeholders, what might be seen as a limitation of the SWOT methodology for analysing chronic care at the system level. As opportunities and threats are linked to external factors and informants were asked to discuss at the system level, it seems logic that it was hard to identify elements situated outside the boundaries of this system.

## Conclusion

This qualitative study with stakeholders working with chronic diseases at the micro and meso level allowed us to formulate some important building blocks for a future health care system oriented towards the needs of patients with chronic conditions. Further research will clarify how the Belgian policy makers can implement the findings of this stakeholders’ analysis. The methodology used in this research could inspire other countries faced with the challenge of drafting reforms to take up the challenge of chronic care.

## Competing interests

The authors declare that they have no competing interests.

## Authors’ contributions

All authors contributed equally to the study design, data collection and data analysis. TVD wrote the draft of the paper and all other authors reviewed and approved the final version of the paper. All authors read and approved the final manuscript.

## Pre-publication history

The pre-publication history for this paper can be accessed here:

http://www.biomedcentral.com/1472-6963/14/179/prepub
